# Hypoxia induces triglycerides accumulation in prostate cancer cells and extracellular vesicles supporting growth and invasiveness following reoxygenation

**DOI:** 10.18632/oncotarget.4479

**Published:** 2015-06-15

**Authors:** Isabel R. Schlaepfer, Dhanya K. Nambiar, Anand Ramteke, Rahul Kumar, Deepanshi Dhar, Chapla Agarwal, Bryan Bergman, Michael Graner, Paul Maroni, Rana P. Singh, Rajesh Agarwal, Gagan Deep

**Affiliations:** ^1^ Division of Medical Oncology, Department of Medicine, University of Colorado Denver, Aurora, Colorado, USA; ^2^ Department of Pharmaceutical Sciences, Skaggs School of Pharmacy and Pharmaceutical Sciences, University of Colorado Denver, Aurora, Colorado, USA; ^3^ Cancer Biology Laboratory, School of Life Sciences, Jawaharlal Nehru University, New Delhi, India; ^4^ Department of Molecular Biology and Biotechnology, Tezpur University, Tezpur, India; ^5^ University of Colorado Cancer Center, University of Colorado Denver, Aurora, Colorado, USA; ^6^ Division of Endocrinology, Metabolism and Diabetes, University of Colorado Denver, Aurora, Colorado, USA; ^7^ Department of Neurosurgery, University of Colorado Denver, Aurora, Colorado, USA; ^8^ Department of Surgery, University of Colorado Denver, Aurora, Colorado, USA

**Keywords:** hypoxia, extracellular vesicle, prostate cancer, lipids, β-oxidation

## Abstract

Hypoxia is an independent prognostic indicator of poor outcome in several malignancies. However, precise mechanism through which hypoxia promotes disease aggressiveness is still unclear. Here, we report that under hypoxia (1% O_2_), human prostate cancer (PCA) cells, and extracellular vesicles (EVs) released by these cells, are significantly enriched in triglycerides due to the activation of lipogenesis-related enzymes and signaling molecules. This is likely a survival response to hypoxic stress as accumulated lipids could support growth following reoxygenation. Consistent with this, significantly higher proliferation was observed in hypoxic PCA cells following reoxygenation associated with rapid use of accumulated lipids. Importantly, lipid utilization inhibition by CPT1 inhibitor etomoxir and shRNA-mediated CPT1-knockdown significantly compromised hypoxic PCA cell proliferation following reoxygenation. Furthermore, COX2 inhibitor celecoxib strongly reduced growth and invasiveness following hypoxic PCA cells reoxygenation, and inhibited invasiveness induced by hypoxic PCA EVs. This establishes a role for COX2 enzymatic products in the enhanced PCA growth and invasiveness. Importantly, concentration and loading of EVs secreted by PCA cells were significantly compromised under delipidized serum condition and by lipogenesis inhibitors (fatostatin and silibinin). Overall, present study highlights the biological significance of lipid accumulation in hypoxic PCA cells and its therapeutic relevance in PCA.

## INTRODUCTION

Prostate cancer (PCA) is the most common non-cutaneous cancer in men. According to the American Cancer Society reports, 233,000 new cases and 29,480 deaths from PCA were estimated in the United States in 2014 [1]. Several studies have shown that low oxygen in the tumor microenvironment, known as hypoxia, determines disease aggressiveness, and is an independent prognostic indicator of poor outcome in PCA as well as in several other malignancies [2–6]. Hypoxic environment within the prostate is considered responsible for the generation of secondary mutations in the genome, leading to aggressive and lethal PCA [7]. Hypoxia is also considered the first major challenge encountered by the growing mass of neoplastic cells, as cancer cells in hypoxic areas are surrounded by metabolic waste, acidic pH and necrotic cells [8, 9]. To overcome these hostile conditions, tumor cells activate transcriptional machinery (e.g. hypoxia inducible factors [HIFs]) leading to neo-angiogenesis and altered anaerobic metabolism; however, the precise mechanism through which hypoxic conditions promote growth and metastasis are still not well defined.

Recent literature has established a critical role for nano-sized vesicles in inter-cellular communication, primary tumor growth, angiogenesis, pre-metastatic niches preparation, metastasis, drug resistance, immunosuppression and disease relapse [10–13]. In the past, ambiguity about the nomenclature of these vesicles resulted in different terms such as, exosomes, microvesicles, ectosomes, shedding vesicles, or microparticles. It is now agreed to label these vesicles as extracellular vesicles (EVs), which are further sub-categorized into exosomes and microvesicles mainly based upon their site of origin; vesicles formed in multi-vesicular bodies (MVBs) of the endosomal system, but released extracellular, are termed exosomes (~ size 50-150 nm), while vesicles budding directly from the membranes are named microvesicles (~ size 100-1000 nm). Recent studies have clearly established that hypoxic cancer cells secrete copious amounts of EVs, which promote tumor growth and progression [12, 14]. King *et al.* showed that breast cancer cells secrete higher amount of exosomes under hypoxic condition in a HIF1α-dependent manner [14]. Under hypoxic conditions, adipocyte-released exosomes promoted lipogenesis in 3T3-L1 recipient cells [15]. Furthermore, exosomes secreted by hypoxic cancer cells promote angiogenesis *via* cargo loaded in these exosomes such as miRNAs [10, 11]. Besides exosomes, hypoxia significantly enhanced the microvesicles biogenesis in a HIF1α- and RAB22A GTPase-dependent manner, and promoted the formation of focal adhesions, invasiveness and metastasis in naïve breast cancer cells [12]. We have recently reported that EVs secreted by PCA cells under hypoxic conditions induce epithelial-to-mesenchymal transition (EMT), invasiveness and stemness in naïve PCA cells; they also promote cancer-associated fibroblast (CAF) phenotype in naïve normal prostate fibroblasts [2]. We have also reported that hypoxic PCA EVs are loaded with unique proteins with higher expression of canonical markers, MMPs, and signaling molecules compared to normoxic PCA EVs [2]; however, little is known about the effect of hypoxia on lipid levels in PCA cells as well as their EVs.

In a recent study, Llorente *et al.* reported that PCA cells exhibit a strong ( > 8-fold) enrichment of lipids in exosomes [16]. The majority of the lipid classes studied in exosomes includes phospholipids, cholesterol and sphingomyelin. However, less attention has been focused on the triglycerides, a form of neutral lipid that is abundant in lipid droplets inside the cells [17]. Triglycerides have been historically regarded as inert lipid depots that accumulate in the cytoplasm of most cells. More recently, the dynamics of lipid droplet triglycerides has been revamped with several studies suggesting that triglyceride stores are dynamic and provide survival benefits to normal and cancer cells [18–20]. In this regard, the present study examined the biological significance of lipid accumulation in PCA cells under hypoxic conditions, as well as the role of EVs as bioactive lipid carriers. Our results show that lipid accumulation under hypoxia supports PCA growth and invasiveness following reoxygenation, and hypoxic PCA EVs loaded with bioactive lipids also enhance the invasiveness of naïve PCA cells. Moreover, these results suggest that EV biogenesis and cargo are dependent upon lipid availability and *de novo* lipogenesis in PCA cells.

## RESULTS

### Hypoxia influences the fatty acid composition of PCA cells and their EVs

LNCaP cells were exposed to normoxic (21% O_2_) or hypoxic (1% O_2_) conditions for 48 hrs. Thereafter, cells and EVs were collected and subjected to lipid isolation and analysis by GC-MS. We examined the fatty acid composition of the phospholipid, triglyceride and diacylglycerol lipid fractions. Table 1A shows the fatty acid composition of phospholipids, triglycerides and diacylglycerols from LNCaP cells and their respective EVs grown under normoxic conditions. Four replicates were used for each sample, and the order of the fatty acids is based on their carbon chain length from top to bottom. Myristic acid (14:0) and palmitic acid (16:0) were significantly decreased in the phospholipids of normoxic LNCaP cells EVs (*p* < 0.05), while stearic acid (18:0) was increased by ~2 fold (*p* < 0.01) and accounts for the increased saturation index of EVs compared to LNCaP cell phospholipids (~70% *vs.* 56.7%, *p* < 0.01). We also observed a significant decrease in oleic acid (18:1) and an increase in linoleic acid (18:2) in the phospholipids of EVs from normoxic LNCaP cells (Table 1A). These data are in agreement with previous studies where palmitic, stearic and oleic acids were the most abundant fatty acids in normoxic exosomes from rodent mast cells [21].

**Table 1:** Lipid content in PCA cells and EVs under normoxic and hypoxic conditions LNCaP cells were cultured under normoxic and hypoxic conditions for 48 hrs. At the end, cells and EVs were collected and analyzed by GC-MS for fatty acid composition (% mol) of phospholipid, triglycerides and diacylglyerols. In each case, 4 biological replicates were analyzed and data are presented as mean±SD.

A	Normoxic conditions (*n* = 4 for each sample type)											
	Phospholipids (% mol)				Triglycerides (% mol)				Diacylglycerols (% mol)			
	Cells		EVs		Cells		EVs		Cells		EVs	
**Fatty acid**	Mean	± SD	Mean	± SD	Mean	± SD	Mean	± SD	Mean	± SD	Mean	± SD
Myristic	1.05	0.2	0.52^$^	0.26	2.9	0.78	6.9	4.2				
Palmitic	30.69	2.2	25.80^$^	3.38	34.1	3.51	31.08	8.73	31.08	1.74	51.21	20.52
Palmitoleic	2.27	0.13	1.71	1.24	1.88	0.3	13.99	13.09				
Stearic	20.73	3.19	41.97^#^	2.57	16.37	0.68	9.80^#^	1.7	37.81	5.62	45.81	18.13
Oleic	40.68	4.4	23.32^#^	1.25	39.75	4.28	15.13^#^	3.82	29.57	6.23		
Linoleic	0.94	0.11	2.18^#^	0.44	1.14	0.21	5.14^#^	1.73				
Arachidonic	0.22	0.03	4.51	4.16	3.86	0.79	17.96^#^	5.99				
**% Saturation**	56.76	2.31	69.99^#^	2.74	55.24	5.15	61.77	8.02	69.62	6.97	97.02^#^	5.96

B	Hypoxic conditions (*n* = 4 for each sample type)											
	Phospholipids (% mol)				Triglycerides (% mol)				Diacylglycerols (% mol)			
	Cells		EVs		Cells		EVs		Cells		EVs	
**Fatty acid**	Mean	± SD	Mean	± SD	Mean	± SD	Mean	± SD	Mean	± SD	Mean	± SD
Myristic	1.23	0.28	0.36^#^	0.21	2.98	1.04	30.43	34.94				
Palmitic	31.28	4.19	26.52	3.08	37.07	1.58	29.92	17.94	44.65	5.33	42.74	11.47
Palmitoleic	1.49	0.17	0.52^#^	0.18	1.37	0.19	3.33	2.78				
Stearic	24.53	1.04	45.48^#^	4.77	22.19	0.62	7.70^$^	4.48	37.12	13	51.47	9.54
Oleic	34.49	4.3	21.73^#^	0.73	25.42	2.07	11.70^$^	8.4	16.52	7.92		
Linoleic	3.11	0.42	2.24^$^	0.34	3.37	0.32	4.62	2.85				
Arachidonic	0.16	0.04	3.15	4.32	7.6	0.51	12.29	7.78				
**% Saturation**	60.89	2.16	72.88^#^	3.97	63.61	2.28	71.39	13.4	81.77	9.71	94.22	7.05

# *p* ≤ 0.01

$ *p* ≤ 0.05

The center column in Table 1A contains the triglyceride information. We found that stearic and oleic acids were significantly decreased in EVs triglycerides compared to the parental cells (*p* < 0.01). Interestingly, the omega-6 fatty acids linoleic acid (18:2) and arachidonic acid (20:4) were strongly represented in the EVs triglycerides (~ 5-fold, *p* < 0.01) underscoring the role of EVs as bioactive lipid carriers. The diacylglycerol fractions of LNCaP cells and EVs did not show any significant changes in fatty acid composition although we only observed the saturated palmitic (16:0) and stearic (18:0) species in EVs (Table 1A).

Table 1B shows the fatty acid composition from LNCaP cells and their respective EVs grown under hypoxic conditions. The percentage of fatty acid saturation in phospholipids was significantly increased in EVs compared to their parental cells (~73% *vs.* ~61% *p* < 0.01), likely driven by a significant 2-fold increase in stearic acid (saturated, *p* < 0.01) and a concomitant decrease in oleic acid (monounsaturated fatty acid, *p* < 0.01). Exposure to hypoxia did not further significantly increase the saturation index of EVs but it did so in the cell membrane phospholipids (~57% saturation in normoxic LNCaP cells *vs*. 61% saturation in hypoxic LNCaP cells, *p* < 0.01). The triglyceride composition in hypoxic conditions showed strong variability in our samples and it was not possible to detect significant changes in fatty acid composition of the EVs, except for stearic and oleic acid species that were significantly reduced (*p* < 0.05). Similar to normoxic condition, we did not observe any significant change in diacylglycerol fractions of LNCaP cells and EVs under hypoxic condition.

**Figure 1:**
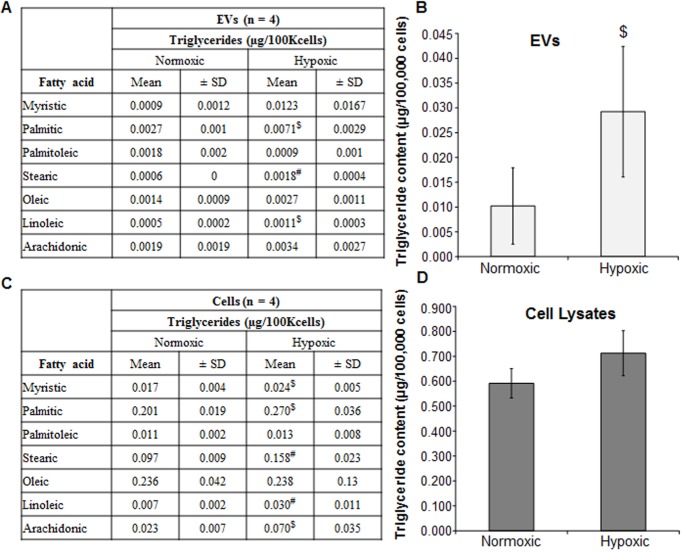
Hypoxia induces triglyceride accumulation in LNCaP cells and EVs **A.**–**D.** LNCaP cells were cultured under normoxic (21% O_2_) and hypoxic (1% O_2_) conditions for 48 hrs. At the end, EVs and cells were collected and analyzed for fatty acid constitution of triglycerides as well as triglyceride content (bar diagrams) and presented as μg/100K cells. In each case, 4 samples were processed and data are presented as mean±SD. #, *p* ≤ 0.01; $, *p* ≤ 0.05.

Further examination of the fatty acid concentration of the triglycerides provided additional information about the changes in the cells and their respective EVs. Figure 1A shows fatty acid composition of triglycerides isolated from EVs of LNCaP cells grown in normoxic and hypoxic conditions. Significant increases in palmitic, stearic and linoleic acids were observed in EVs^Hypoxic^ compared to EVs^Normoxic^ (~2-fold, *p* < 0.01). Overall, a 3-fold increase in triglycerides was observed under hypoxia when compared to normoxia (*p* < 0.05) (Figure 1B). In LNCaP cells, myristic, palmitic and stearic fatty acids (all saturated fatty acids) were significantly increased under hypoxia compared to normoxia (Figure 1C). Of note, essential linoleic acid and its derivative arachidonic acid were both significantly increased in the triglycerides of hypoxic LNCaP cells (4.5-fold and 3-fold, respectively; *p* < 0.05) (Figure 1C). These results are important since both fatty acids are strongly implicated in inflammation and malignant tumor growth. Similar to EVs^Hypoxic^, LNCaP cells also showed higher triglycerides level under hypoxic condition, but the changes were not significant (Figure 1D).

### Altered signaling and metabolic enzymes expression in LNCaP cells exposed to hypoxia

**Figure 2:**
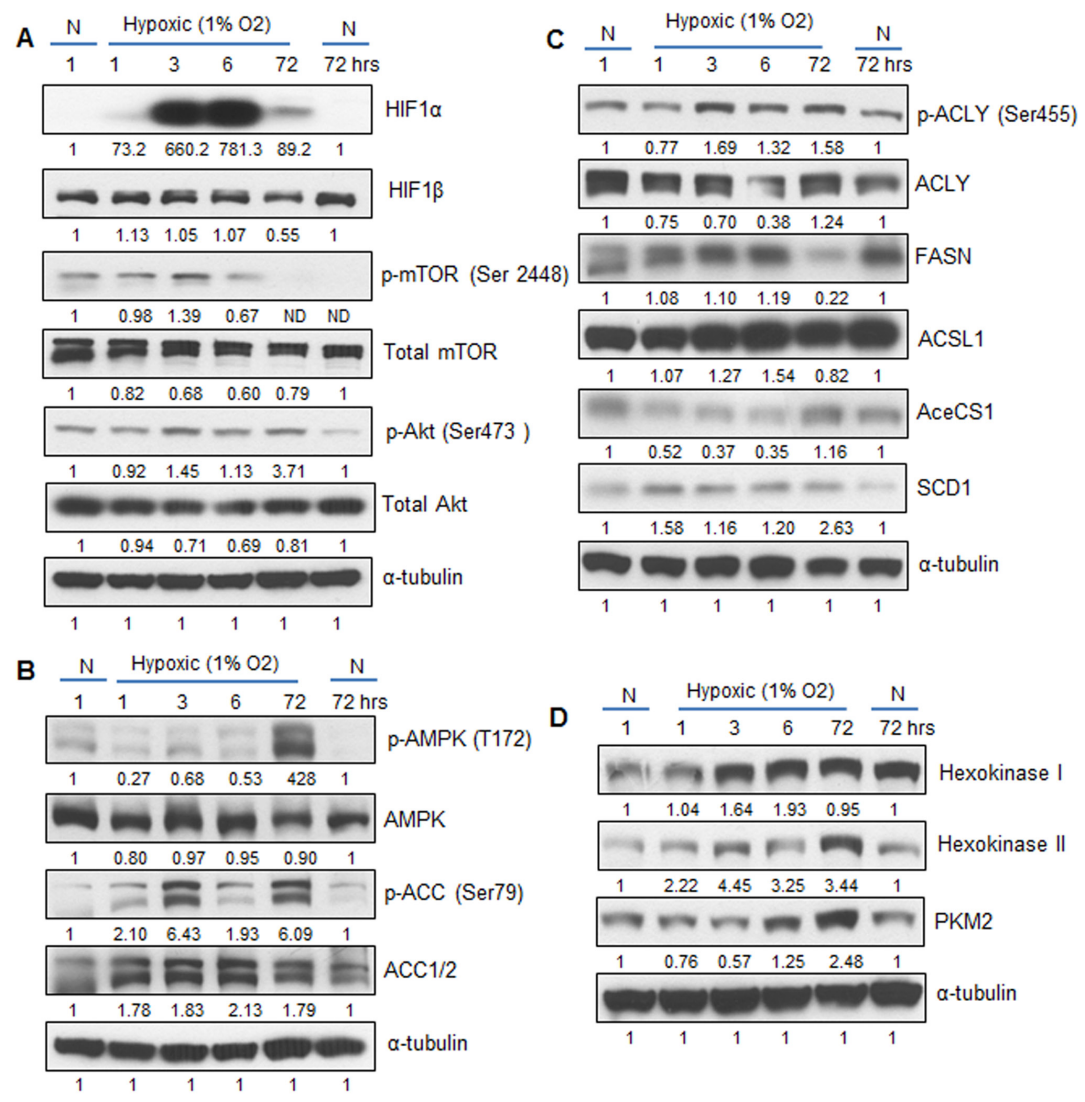
Hypoxia activates signaling cascades involved in lipid synthesis LNCaP cells were exposed to hypoxia (1% O_2_) and cell lysates were prepared after 1, 3, 6 and 72 hrs. Cells cultured under normoxic condition (21% O_2_) at 1 and 72 hrs served as relevant controls. Cell lysates were prepared and analyzed by immunoblotting for the expression of various **A.** signaling molecules, **B.**–**C.** lipogenesis regulators, and **D.** glucose metabolism enzymes. Membranes were stripped and re-probed for α-tubulin and representative blots are shown. Densitometry data presented below the bands are ‘fold change’ as compared with respective normoxic control after normalization with respective loading control (α-tubulin). N: Normoxic; ND: Not detectable.

In order to investigate the molecular changes in LNCaP cells exposed to hypoxia, we collected cell lysates following 1, 3, 6 and 72 hrs of hypoxia exposure, and at 1 and 72 hrs of normoxia exposure (first and last lanes, Figure 2). These lysates were used to interrogate expression of signaling molecules and enzymes involved in lipid and glucose metabolism. Figure 2A shows the strong expression levels of HIF1α within 3 hrs of exposure to hypoxia. Phosphorylation of mTOR (mammalian target of rapamycin) was also increased by 3 hrs, concomitant with the Akt activation (Figure 2A). The phosphorylation of mTOR decreased rapidly by 6 hrs in hypoxia and no expression was observed at 72 hrs. This could be partly due to longer incubation time of the cells that depletes the media of nutrients, since cells grown in normoxic conditions for 72 hrs (last lane) also did not show the activation of mTOR (phosphorylation at Serine 2448) (Figure 2A). However, activation of Akt (Ser 473) was still observed after 72 hrs of hypoxia (Figure 2A).

Since inactivation of mTOR was observed at later time-point under hypoxia, we then examined the expression and activation status of the cellular fuel sensor AMPK (AMP-activated protein kinase) and one of its downstream targets- ACC (acetyl Co-A carboxylase). As shown in Figure 2B, AMPK was activated at 72 hrs time-point under hypoxia. This is in accordance with earlier reports, which showed that hypoxia induces AMPK levels very late [22, 23]. This could be explained by the fact that AMPK activation is dependent on the AMP/ATP ratio, and in the initial stages of hypoxia, cellular ATP levels are maintained via enhanced glycolysis. Hence, AMPK becomes active only at late stages. The ACC enzyme uses glucose carbons to generate malonyl-coA that leads to *de novo* lipid synthesis. Phosphorylation of ACC at Serine 79 by AMPK results in inactivation of the enzyme. We observed an increase in ACC phosphorylation along with higher total ACC protein (~ 2-fold) under hypoxia (Figure 2B), suggesting high carboxylase activity at 6 hrs leading to fatty acid synthesis.

Figure 2C shows the expression of lipid synthesis enzymes (ATP-citrate lyase, acetyl-CoA synthetase and fatty acid synthase) and post-synthesis enzymes (acyl CoA synthase ligase, and steroyl-CoA-desaturase1) under hypoxic condition. The ATP-citrate lyase (ACLY) enzyme is activated by phosphorylation at Serine 455 by Akt. Activation of the ACLY enzyme at 3, 6 and 72 hrs parallels the activation of Akt in Figure 2A. The fatty acid synthase enzyme (FASN) expression increased under hypoxia but was strongly decreased by 72 hrs (Figure 2C), matching the inactivation of ACC by AMPK (Figure 2B). Furthermore, the enzyme that activates newly synthesized fatty acids, acyl CoA synthase ligase (ACSL) also showed an increase at early time points. This is relevant since the newly synthesized fatty acids need to be processed by ACSL in order to be directed to membrane phospholipids or triglycerides. Note the low expression of acetyl-CoA synthetase (AceCS1) in hypoxia, suggesting that acetate is not the main substrate for the lipid synthesis. Another enzyme involved in lipid synthesis is steroyl-CoA desaturase (SCD1), which is involved in the generation of monounsaturated fatty acids (like oleic and palmitoleic acid) [24]. However, little is known about the mechanisms of SCD1 action in PCA cells under hypoxia. We observed increased expression of SCD1 in hypoxic cells from 1 to 72 hrs (Figure 2C), suggesting a protective response, since accumulation of saturated lipids by inhibition of SCD1 promotes cell death [25]. Overall, these molecular results add strength to the lipogenic phenotype of PCA cells grown under hypoxic conditions.

Activation of HIF1α by hypoxia is known to be associated with a glycolytic phenotype in many cancer cells, with a substantial increase in glucose uptake. We next examined the expression of hexokinases and pyruvate kinase 2 (muscle isoform or PKM2) as surrogates of increased glucose uptake in the hypoxic LNCaP cells. Figure 2D shows that both hexokinases (I and II) are upregulated under hypoxia. Hexokinase is most abundant in cancer cells and is a direct target of HIF1α protein, a result that correlates with the HIF1α activation shown in Figure 2A. Pyruvate kinase 2 is also a master regulator of metabolism in cancer cells [26]. It is upregulated by HIF1α and modulates the production of lactate and glycolytic intermediates necessary for lipid and nucleic acid synthesis. A gradual increase in PKM2 between 3 and 72 hrs was observed in hypoxic LNCaP cells (Figure 2D). These results suggest that increased glucose uptake in hypoxia promotes *de novo* lipogenesis in PCA cells mediated by ACLY, ACC and FASN enzymes (Figure 2C).

### Hypoxia-induced lipid accumulation promotes proliferation in PCA cells following reoxygenation

**Figure 3:**
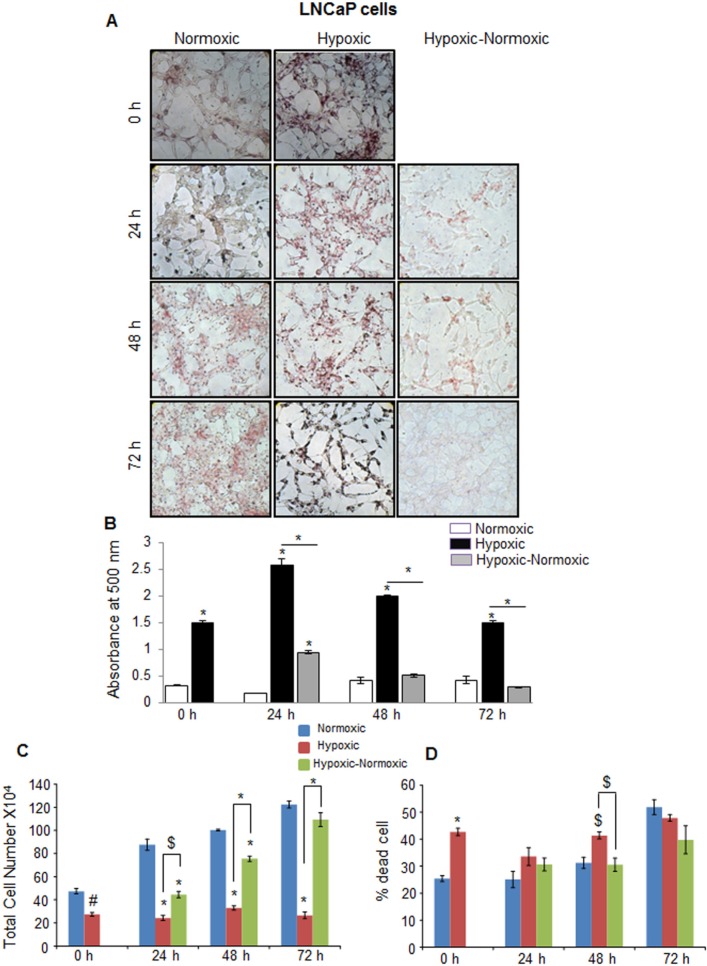
Hypoxia induces lipid accumulation and promotes proliferation following reoxygenation in prostate cancer LNCaP cells LNCaP cells were seeded at 50,000 cells/well in a 6 well plate. After 36 hrs, media was changed and cells were either transferred to hypoxia chamber (1% O_2_) or continued under normoxia (21% O_2_). After 48 hrs, one set of hypoxic plates was transferred to normoxia and maintained in normoxic conditions for 24, 48 and 72 hrs, while another set remained under hypoxia. **A.**–**B.** After completing the respective time periods, cells were fixed with 10% formaldehyde and stained with ORO. Representative pictures (at 200X) for ORO staining are shown. Lipid content quantitation was carried out by dye elution using isopropanol, and absorbance was measured by spectrophotometer at 500 nm. **C.**–**D.** At every time-point, total cell number and dead cell percentage were also measured. *, *p* ≤ 0.001; #, *p* ≤ 0.01; $, *p* ≤ 0.05.

We next examined the role of lipid stores on PCA growth following reoxygenation. PCA cells were cultured under hypoxic conditions for 48 hrs (labeled as 0 hr), and thereafter, maintained under continuous hypoxia (for 24-72 hrs) or returned to normoxia (for 24-72 hrs). Throughout, cell proliferation rate was measured by trypan blue assay, and correlated with cellular lipid level by staining cells with Oil red O (ORO) that mainly stains cellular neutral triglycerides and cholesteryl esters. As shown in Figure 3A and 3B, at each time-point, there was significantly higher lipid accumulation in hypoxic LNCaP cells compared with normoxic cells. Following reoxygenation, LNCaP cells (labeled ‘Hypoxic-Normoxic’) showed a consistent decrease in accumulated lipid level compared with hypoxic cells (Figure 3A and 3B). We speculated that under hypoxic stress, PCA cells store energy in the form of lipid droplets, which would be burned when a favorable environment (O_2_) is available. As expected, under hypoxic conditions, LNCaP cell growth was significantly inhibited and higher cell death was observed at most of the time-points (Figure 3C and 3D). However, following reoxygenation, LNCaP cell proliferation increased steadily (Figure 3C). Interestingly, growth rate of hypoxic-normoxic cells was even higher than normoxic LNCaP cells (Figure 3C). Regarding cell death, at 72 hrs time point, the hypoxic-normoxic cells showed 38% cell death, while the normoxic and hypoxic controls showed 52 and 48% cell respectively (Figure 3D). Notably, the increase in cell number in hypoxic-normoxic group correlated inversely to the lipid content.

**Figure 4:**
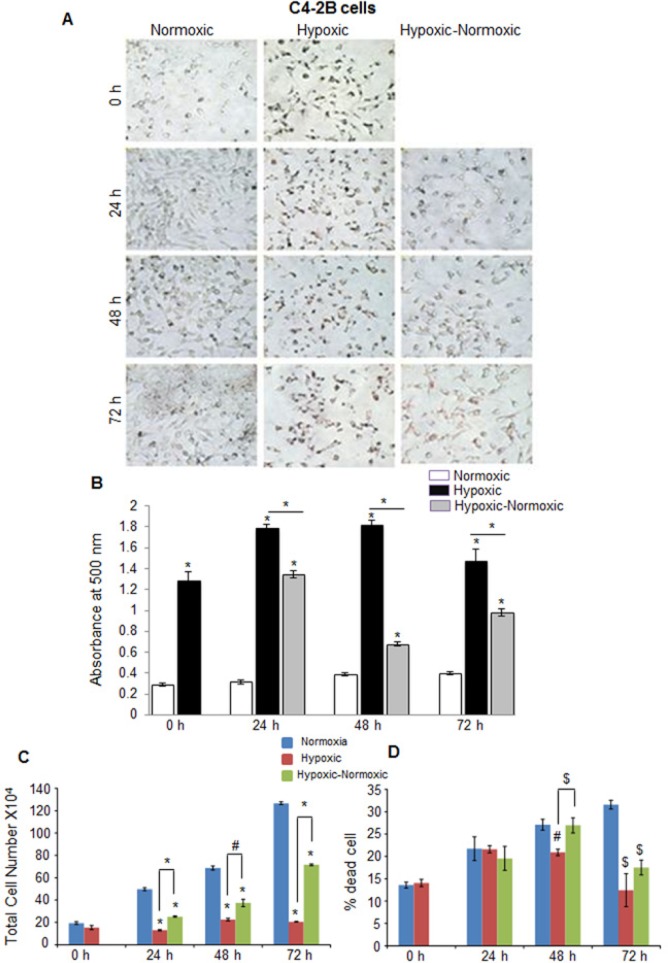
Hypoxia induces lipid accumulation and promotes proliferation following reoxygenation in prostate cancer C4-2B cells C4-2B cells were seeded at 50,000 cells/well in a 6 well plate. After 36 hrs, media was changed and cells were either transferred to hypoxia chamber (1% O_2_) or continued under normoxia (21% O_2_). After 48 hrs, one set of hypoxic plates was transferred to normoxia and maintained in normoxic conditions for 24, 48 and 72 hrs, while another set remained under hypoxia. **A.**–**B.** After completing the respective time periods; cells were fixed with 10% formaldehyde and stained with ORO. Representative pictures (at 200X) for ORO staining are shown. Lipid content quantitation was carried out by dye elution using isopropanol, and absorbance was measured by spectrophotometer at 500 nm. **C.**–**D.** At every time-point, total cell number and dead cell percentage were also measured. *, *p* ≤ 0.001; #, *p* ≤ 0.01; $, *p* ≤ 0.05.

**Figure 5:**
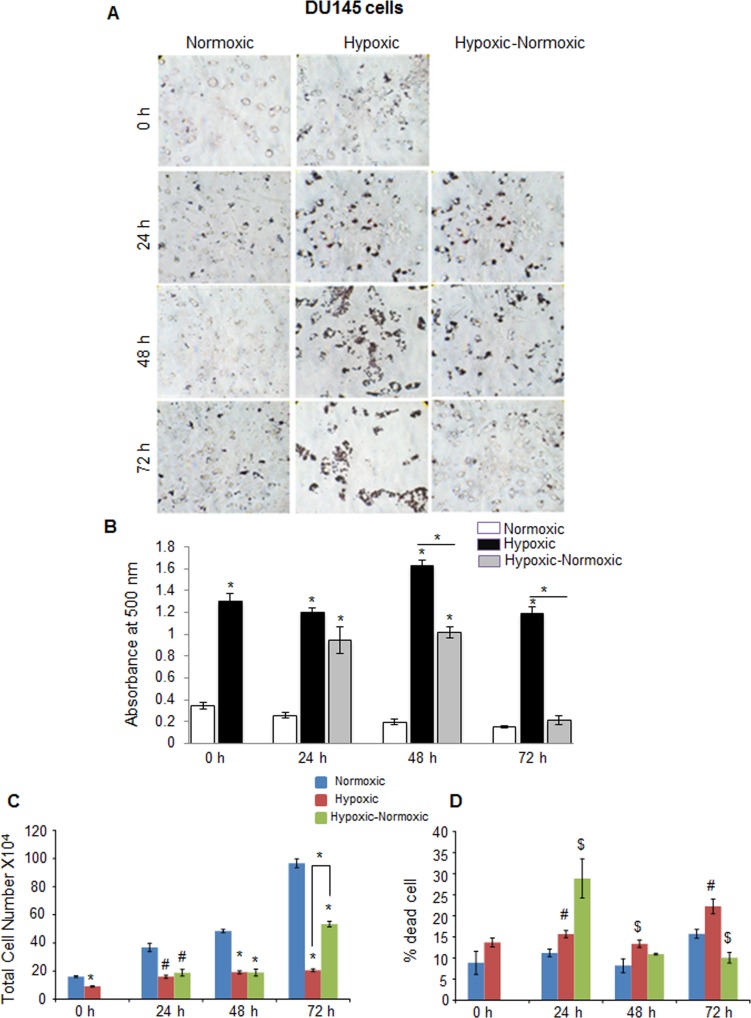
Hypoxia induces lipid accumulation and promotes proliferation following reoxygenation in prostate cancer DU145 cells DU145 cells were seeded at 50,000 cells/well in a 6 well plate. After 36 hrs, media was changed and cells were either transferred to hypoxia chamber (1% O_2_) or continued under normoxia (21% O_2_). After 48 hrs, one set of hypoxic plates was transferred to normoxia and maintained in normoxic conditions for 24, 48 and 72 hrs, while another set remained under hypoxia. **A.**–**B.** After completing the respective time periods; cells were fixed with 10% formaldehyde and stained with ORO. Representative pictures (at 200X) for ORO staining are shown. Lipid content quantitation was carried out by dye elution using isopropanol, and absorbance was measured by spectrophotometer at 500 nm. **C.**–**D.** At every time-point, total cell number and dead cell percentage were also measured. *, *p* ≤ 0.001; #, *p* ≤ 0.01; $, *p* ≤ 0.05.

In C4-2B (an androgen-independent LNCaP subline) and DU145 PCA cells, we observed rather similar responses to hypoxia in terms of higher neutral lipid accumulation as well as proliferation following reoxygenation (Figure 4 and Figure 5). However, C4-2B cells took much longer time than LNCaP cells to utilize the lipid reserves and grow aggressively (Figure 4A and 4B). We found that after 24 hrs of shifting the cells to normoxia, the lipid content was reduced by 23%, but by the end of 48 hrs, the lipid content was decreased by 67% (Figure 4B). The cell number also increased significantly especially at the end of 72 hrs, but surprisingly, we observed relatively higher death in reoxygenated cells compared with hypoxic cells (Figure 4C and 4D). A similar trend was observed in DU145 cells, with maximum decrease in lipid accumulation at 72 hrs following reoxygenation (Figure 5A and 5B). DU145 cells in normoxic condition grew by 2.4 times between 24 and 72 hrs time-points; in hypoxic condition, we did not see any significant growth; however following reoxygenation, the cells grew by 2.7 times between 24 and 72 hrs time-points (Figure 5C and 5D). The percentage of cell death also showed a significant decrease in hypoxic-normoxic condition compared with normoxia or hypoxia at 72 hrs after reoxygenation (Figure 5D).

### Inhibition of lipid β-oxidation compromised the cell growth following reoxygenation in hypoxic LNCaP cells

**Figure 6:**
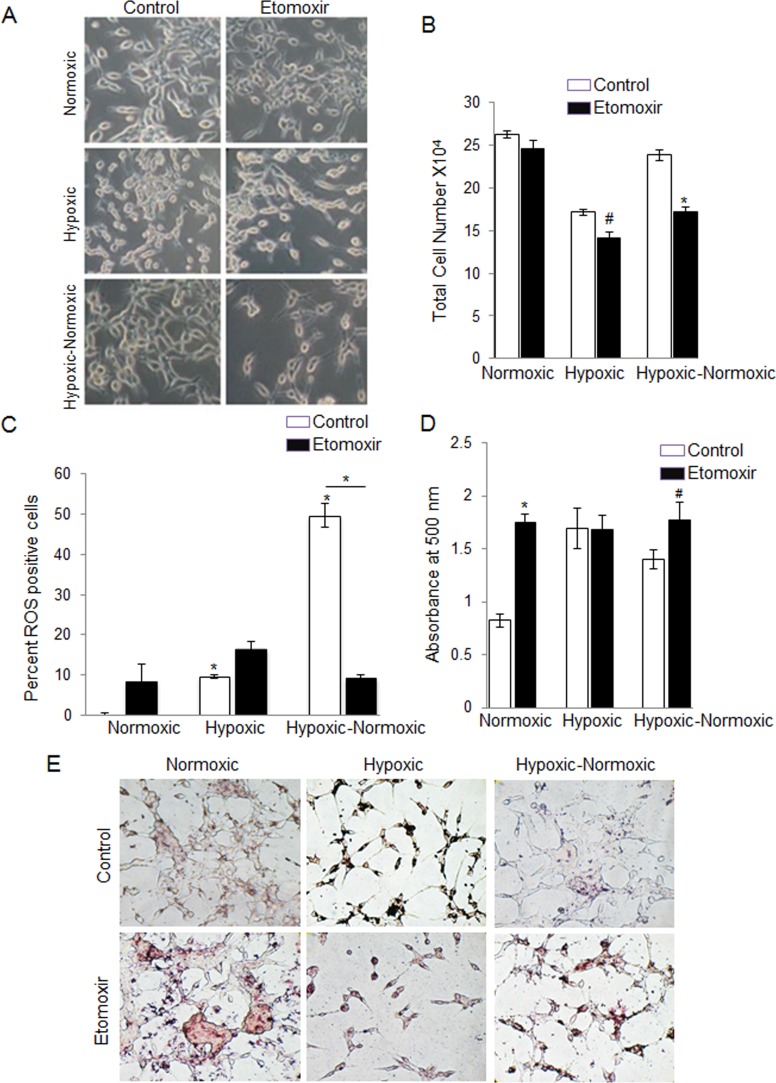
Inhibition of lipid β-oxidation by etomoxir decreases cell growth following reoxygenation in hypoxic LNCaP cells LNCaP cells were seeded at 50,000 cells/well in a 6 well plate. After 36 hrs, media was changed and cells were either transferred to the hypoxia chamber (1% O_2_) or continued in normoxia (21% O_2_). After 48 hrs, one set of hypoxic plates was transferred to normoxia, while another set remained under hypoxic conditions. All the groups (normoxic, hypoxic and hypoxic-normoxic) were treated with etomoxir. After 24 hrs of etomoxir treatment, **A.** images were captured for cell morphology; **B.** total cell number was measured; **C.** ROS level was measured by flow cytometry as detailed in the methods; and **D.**–**E.** lipid content was measured via ORO staining and quantified; and representative pictures (at 200X) for ORO staining are shown. *, *p* ≤ 0.001; #, *p* ≤ 0.01.

As mentioned above, we observed a striking inverse correlation between reoxygenation and cellular lipid content in the PCA cells. This led us to speculate that the accumulation of lipids during hypoxic conditions allows cells to survive in a cytostatic state until reoxygenation. Upon reoxygenation, these cells could break down the stored lipids by β-oxidation, and use the energy to proliferate. This notion is supported by studies claiming that, under hypoxic conditions, β-oxidation is inhibited, and a recent report suggested that CPT1 and ACSL1, which facilitate fatty acid import and oxidation, respectively, were both suppressed by activation of HIF2α in liver-specific VHL-knockout mouse model [27]. Hence, we decided to use a synthetic β-oxidation inhibitor, etomoxir, to target the growth of these tumor cells upon reoxygenation. Under normoxic conditions, treatment of LNCaP cells with etomoxir did not result in any cytotoxicity (Figure 6A and 6B). However, we observed a moderate decrease in cell number in hypoxic condition (17.6%; *p* < 0.01), but in the case of hypoxic-normoxic cells, the number decreased by 28% (*p* < 0.001), suggesting that fatty acid oxidation plays an important for sustained growth during reoxygenation (Figure 6A–6B).

It is also known that β-oxidation is a major source for intracellular ROS generation [28]. Thus, we anticipated that the reoxygenated cells would show higher amount of intracellular ROS production. We found that under normoxic and hypoxic condition, less than 10% of the cells were ROS positive, but with reoxygenation, there was a drastic increase in the ROS positive population (49.7%; *p* < 0.001) (Figure 6C). Addition of etomoxir during reoxygenation completely reversed this, with only 9.7% cells positive for ROS (Figure 6C).

Additionally, we found a significant increase (48%) in lipid accumulation with etomoxir treatment in LNCaP cells under normoxic condition (Figure 6D and 6E). As expected, under hypoxic conditions, etomoxir treatment did not lead to any change in lipid accumulation, as β-oxidation is already inhibited under these conditions (Figure 6D and 6E). With reoxygenation, addition of etomoxir led to 17% increase in lipid compared to hypoxic-normoxic control (Figure 6D and 6E).

**Figure 7:**
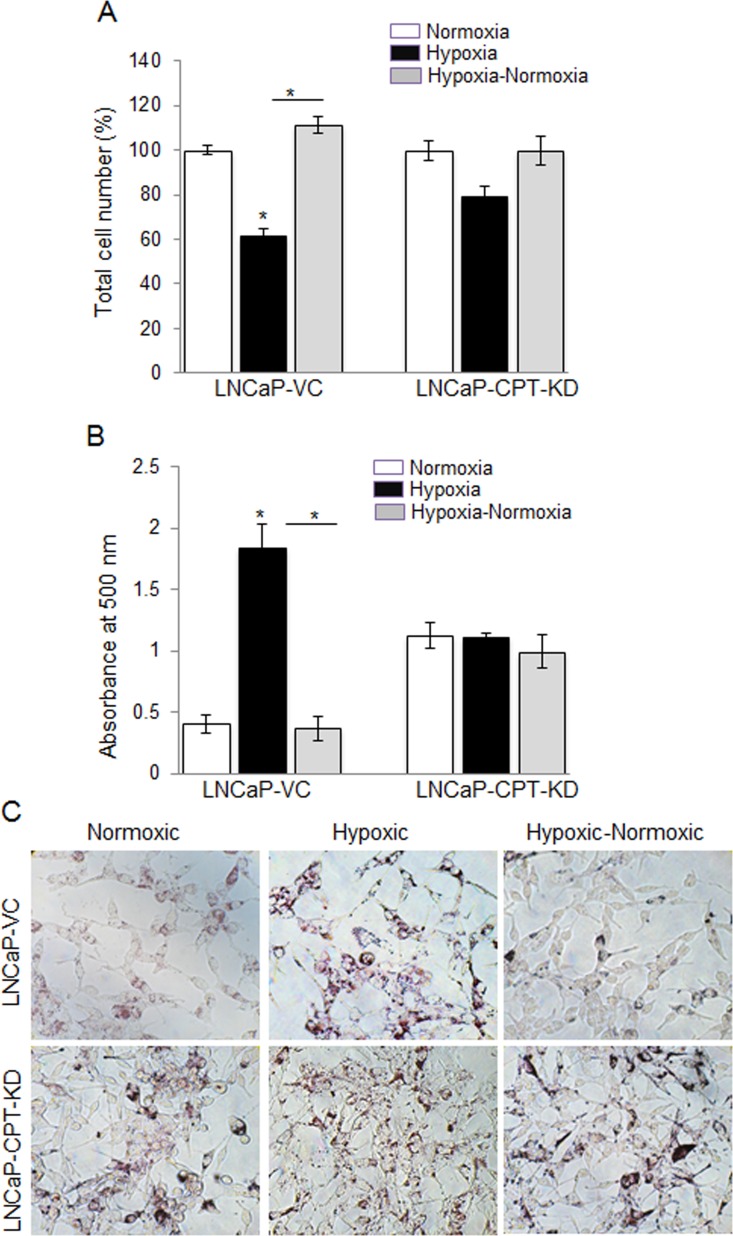
CPT1 knock-down compromised cell growth due to lack of lipid use following reoxygenation in hypoxic LNCaP cells LNCaP-VC and LNCaP-CPT-KD cells were seeded at 50,000 cells/well in a 6 well plate. After 36 hrs, media was changed and cells were either transferred to the hypoxia chamber (1% O_2_) or continued in normoxia (21% O_2_). After 48 hrs, one set of hypoxic plates was transferred to normoxia, and 24 hrs later **A.** total cell number was counted and presented as percentage relative to 100% normoxic control cells; **B.**–**C.** lipid content was measured via ORO staining and quantified; and representative pictures (at 200X) for ORO staining are shown. *, *p* ≤ 0.001.

To further verify the role of β-oxidation in the reoxygenation-induced growth, we used LNCaP CPT1 knocked down (KD) cells under different oxygen conditions. We have reported earlier that LNCaP-CPT1-KD cells have a 60% decrease in β-oxidation [29]. As expected, in LNCaP vector control (VC) cells, hypoxia caused a 40% decrease in cell number, but in hypoxic-normoxic conditions, the cell number significantly increased (Figure 7A). However, in the LNCaP-CPT1-KD cells, hypoxia exposure resulted in only a slight decrease (~12%) in cell number, and no significant increase in proliferation was observed following reoxygenation of hypoxic cells (Figure 7A). The molecular mechanism responsible for relatively better survival of LNCaP-CPT1-KD cells compared to LNCaP-VC cells under hypoxic conditions is unknown, but could be attributed to higher anaerobic glycolysis, as these cells have shown increased uptake of glucose compared to control cells (VC) [30]. Alternatively, the better survival of LNCaP-CPT1-KD could be due to decreased intracellular ROS generation associated with lower β-oxidation in these cells. Further, as expected, LNCaP-CPT1-KD cells did not show any lipid accumulation under hypoxic conditions compared to normoxic conditions, and compared to vector control cells, showed increased lipid accumulation following reoxygenation, confirming an impediment in the β-oxidation pathway (Figure 7B and 7C).

### Celecoxib blocks hypoxia-mediated clonogenicity of PCA cells

**Figure 8:**
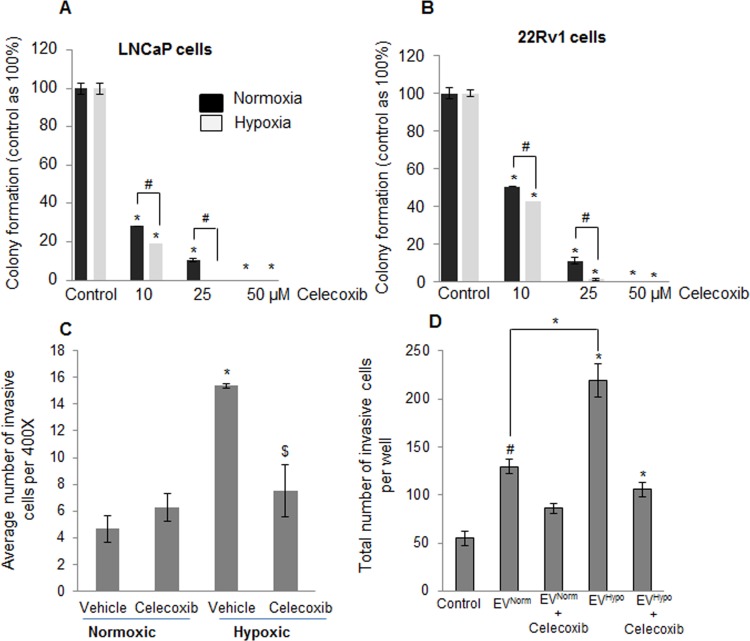
Increased sensitivity of hypoxia-exposed PCA cells towards celecoxib following reoxygenation **A.** LNCaP and **B.** 22Rv1 cells were exposed to hypoxia for 48 hrs and returned to normoxia with or without celecoxib for 6 days. Clones were counted as detailed in the methods and presented as percentage of control. **C.** LNCaP cells were exposed to normoxic or hypoxic conditions for 48 hrs; thereafter cells were collected and analyzed for invasiveness in the presence or absence of celecoxib (25 μM). After 22 hrs, number of invasive cells was determined for each group as detailed in the methods. **D.** EV^Normoxic^ and EV^Hypoxic^ were collected from LNCaP cells as detailed in methods. Thereafter, naïve LNCaP cells (100,000 cells per well) were incubated along with 25 μg of EV^Normoxic^ or EV^Hypoxic^ in the presence or absence of celecoxib (25 μM) and invasiveness was measured after 22 hrs.*, *p* ≤ 0.001; #, *p* ≤ 0.01; $, *p* ≤ 0.05.

As mentioned above, we observed increased levels of arachidonic acid (AA) and linoleic acid (AA precursor) in PCA cells exposed to hypoxia. It is known that metabolism of these lipids through oxygenases (such as COX2) generates potent signaling molecules that promote cell proliferation and invasiveness [31–33]. Therefore, we next examined the effect of the COX2 inhibitor, celecoxib, on the clonogenicity of LNCaP and 22Rv1 cells exposed to hypoxia for 2 days. Figure 8A shows that addition of 10 μM celecoxib to cells resulted in 75% (p ≤ 0.001) decrease in colony formation in normoxia, and 80% (p ≤ 0.001) decrease in clonogenicity under hypoxia. At the 25 μM celecoxib dose, the hypoxia-treated cells did not form any colonies but a few clones were observed under normoxia (Figure 8A). The 22Rv1 cells showed the same pattern as LNCaP cells (Figure 8B). These results suggested the importance of COX2 enzymatic products in the growth of reoxygenated PCA cells.

### Celecoxib blocks the hypoxia-mediated invasive behavior of LNCaP cells

Next, we examined whether the accumulated lipids in LNCaP cells under hypoxic conditions are also involved in invasiveness. We exposed the LNCaP cells to hypoxic conditions for 48 hrs, a time-point by which these cells accumulate lipids. Next, we collected these cells and examined their invasiveness in the presence of celecoxib. As shown in Figure 8C, hypoxia exposure significantly increased the invasive cell population, which was abrogated by celecoxib treatment, suggesting that the invasive phenotype due to the hypoxia is likely mediated by arachidonic acid-derived metabolites *via* COX2 activity. Similarly, EVs collected from hypoxic LNCaP cells (EV^Hypoxic^) increased the invasiveness of LNCaP cells (1.7 fold) compared to EVs collected from normoxic LNCaP cells (EV^Normoxic^) (Figure 8D). More importantly, celecoxib treatment significantly reduced the EV^Hypoxic^-induced invasiveness in LNCaP cells (Figure 8D) further highlighting the role of COX enzymes and arachidonic acid-derived metabolites.

### Lipids are important for EVs synthesis and loading

**Figure 9:**
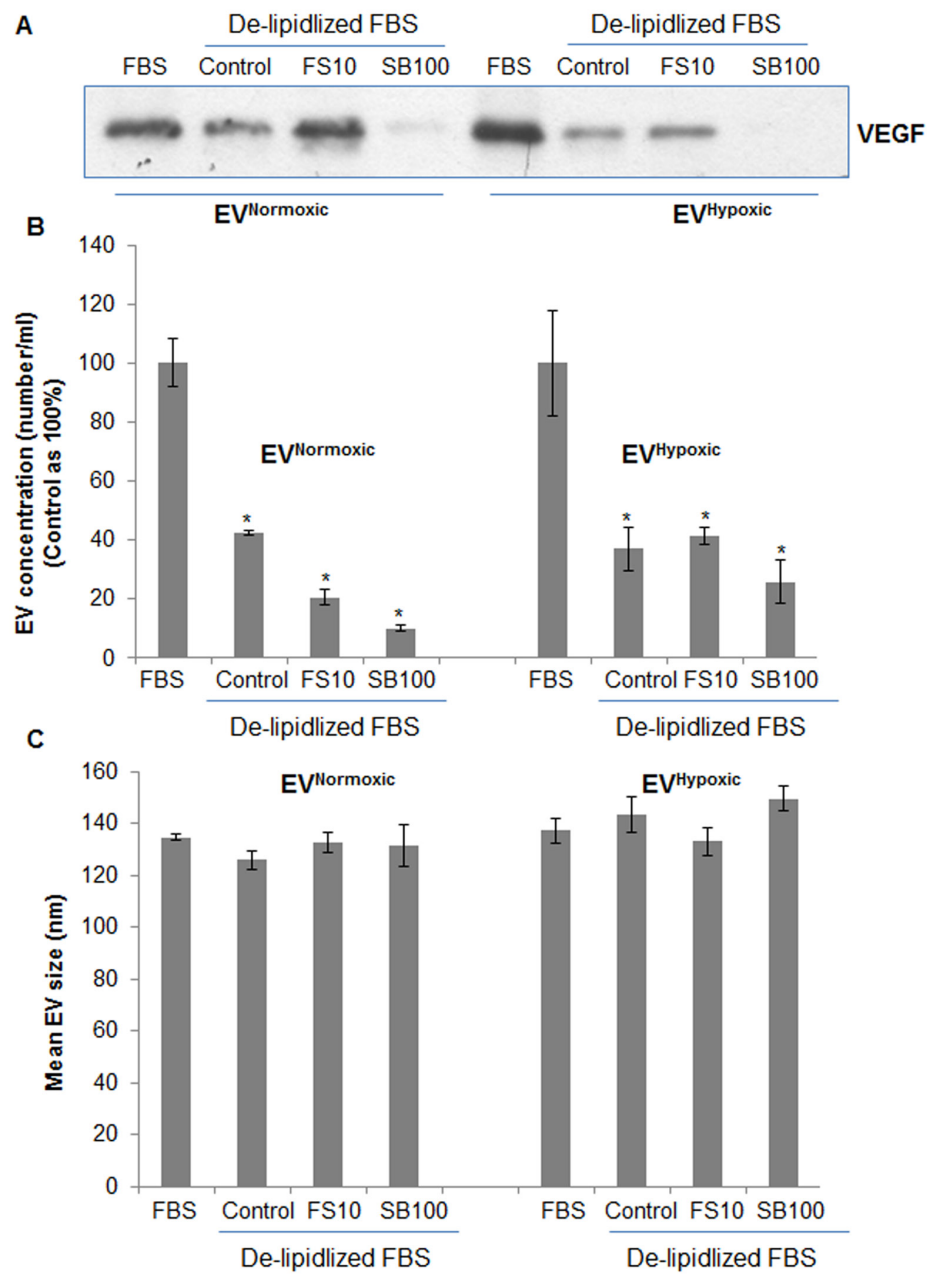
EV^Normoxic^ or EV^Hypoxic^ concentration and loading is compromised in LNCaP cells cultured under reduced lipid conditions and in the presence of lipogenesis inhibitors LNCaP cells were cultured under normoxic and hypoxic conditions either under 10% FBS or delipidized 10%FBS conditions in the presence of fatostatin (10 μM) and silibinin (100 μM). Under each treatment condition, EVs were isolated and analyzed for **A.** VEGF loading by immunoblotting and **B.**–**C.** concentration (number/ml) and mean size by NTA.

Next, we tested whether cellular lipid levels are crucial for EV biosynthesis and loading. LNCaP cells were cultured under FBS or delipidized serum in the presence of lipogenesis inhibitors fatostatin (FS) and silibinin [34]. As shown in Figure 9A and 9B, LNCaP cells under delipidized serum condition secreted significant lesser amount of EVs under both normoxic and hypoxic condition, and also resulted in a significant decrease in VEGF loading in the EVs. Since PCA cells could synthesize lipids under low external lipid conditions, we also treated cells with lipogenesis inhibitor FS or silibinin. As shown in the Figure 9A and 9B, silibinin but not FS showed additional decrease in EV concentration as well as VEGF loading, probably due to its pan-inhibitory effects on lipogenesis [34] compared to the more specific inhibition of SREBP1/2 by FS. Also, we observed relatively higher VEGF loading in EVs by FS treatment especially under normoxic conditions (Figure 9A); the reason and mechanism responsible for that need further examination. Importantly, we did not observe any significant change in the mean EV sizes in any of the group (Figure 9C).

## DISCUSSION

**Figure 10:**
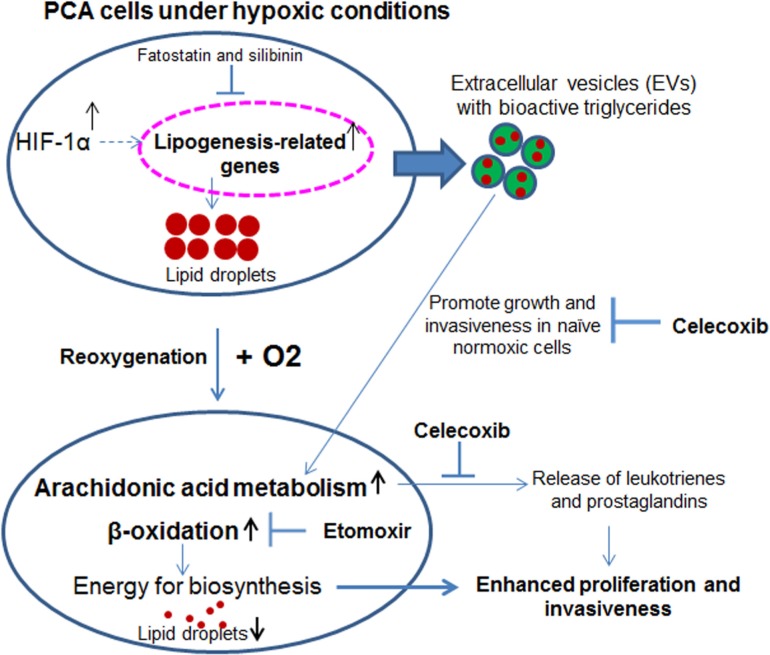
Hypoxia induces PCA aggressiveness *via* enhancing lipid accumulation in cells and EVs Proposed model suggesting that under hypoxic condition lipids are accumulated in cells as well as loaded in EVs. Specific lipid moieties are used as energy sources as well as cues for higher growth and invasiveness when hypoxic cells are reoxygenated, or when normoxic cells receive EV^Hypoxic^. These hypoxia-induced aggressive features could be targeted by drugs such as fatostatin, silibinin, etomoxir and celecoxib.

Hypoxia is a major contributor to tumor growth and its aggressiveness. Typically, hypoxia is detrimental and unfavorable for cell proliferation but cancer cells survive even in harsh hypoxic conditions. The means by which cancer cells adapt to these conditions and sustain their growth remain largely unknown. Previous studies from our lab have identified unique sets of proteins that are loaded in hypoxic PCA EVs and could be responsible for the enhanced invasiveness, stemness and microenvironment changes in PCA cells [2]. The key findings of the present study are: a) under hypoxic conditions, specific lipid moieties are accumulated in PCA cells and their EVs; b) accumulated lipids play an important role in the growth and invasiveness of hypoxic PCA cells following reoxygenation; and c) hypoxia-induced aggressiveness (growth and invasiveness) could be compromised *via* targeting arachidonic acid pathway as well as the lipogenesis and lipid catabolism pathways (Figure 10).

The accumulation of lipids in cells and their EVs under hypoxia is likely derived by the *de novo* synthesis of lipids using glucose carbons from the media. This is corroborated by the fact that ACLY and ACSL1 are upregulated during hypoxia, while the acetate-derived lipid synthesis *via* AceCS1 does not seem to play a role in hypoxia [35]. These results are also in line with earlier reports, which showed hypoxia-stimulated lipid storage and attenuated lipolysis in cardiomyocytes and macrophages [36, 37]. An increase in lipogenesis is also well documented in cancer cells especially in PCA cells [38, 39]; although no prior study has examined the triglyceride accumulation in EVs from hypoxic PCA cells.

Prostate tumors are known to import more fat than glucose compared to other tumors [34, 40]. Hence β-oxidation of lipids is considered an important alternative energy source [41]. Our results implicate the role of lipid catabolism (β-oxidation) in the growth of hypoxic PCA cells especially following reoxygenation. This is important since targeting the ability of cells to burn the lipid is likely a therapeutic window to treat the refractory hypoxic areas of tumors that get exposed to oxygen, like post-surgery or after radiation treatments. Additionally, lipogenesis inhibitors that limit the accumulation of lipid droplets (like silibinin) could synergize with fat burning inhibitors (like etomoxir) to limit the production of substrate available for oxidation. However, further specific tracer studies (using labelled glucose or fatty acids) are required to track the fate of lipids accumulated under hypoxia, helping elucidate if the lipids are used as an energy source and/or for membrane synthesis for growth.

Several lipids such as BMP (bis-monoacylglycerolphosphate), ceramides, cholesterol etc have been reported to play important role in exosome/EV biogenesis and loading [42]. The present study further highlights the role of cellular lipids in the biogenesis of EVs, as concentration of EVs as well as VEGF loading decreased significantly under delipidized serum condition. Moreover, the lipogenesis inhibitor silibinin significantly reduced the EV concentration as well as VEGF loading. These results suggest that EV synthesis as well as EV-regulated signaling in the tumor microenvironment could be targeted by decreasing lipid delivery (via hypolipidemic drugs) as well as reducing the ability of tumors to synthesize lipid (via fatostatin or silibinin) (Figure 10). However, more definite studies are warranted to understand the molecular mechanism underlying the loading of bioactive lipids in EVs especially under hypoxia.

EVs offer a great source of bioactive lipids once they arrive to their final destination (‘recipient cells’) and are processed. Lipids transported by EVs could be delivered to various cytosolic proteins like lipases for triglycerides breakdown. For example, palmitic and oleic fatty acids delivered by hypoxic EVs could be used to generate more phospholipids for membrane synthesis or used as fuel in the mitochondria to generate ATP if the recipient cells is oxygenated (which is the case in the periphery or margins of the tumors). Similarly, other fatty acids transported by EVs, like arachidonic acid can be delivered to enzymes located on intracellular membranes and allow the production of bioactive lipid molecules (eicosanoids) that could enhance growth and motility of the recipient cell. Through these mechanisms, hypoxic PCA EVs could enhance the overall aggressiveness of the tumor mass.

The role of the linoleic acid and its derivative arachidonic acid in the growth and invasive phenotype of PCA is an active area of research. These lipids are substrates for oxygenases (like COX1, COX2 and lipoxygenases) that transform arachidonic acid into potent lipid signaling molecules including prostaglandins, leukotrienes, and hydroxylated forms of arachidonic acid. Our results indicate that hypoxia promotes shunting of arachidonic acid to lipid droplets or triglyceride stores inside cells and EVs, generating a powerful reserve of signaling metabolites. In fact, using the COX inhibitor celecoxib, we were able to decrease the clonogenic and invasive potential of PCA cells that were exposed to hypoxia. We also observed that EVs^Hypoxic^-induced invasiveness is significantly compromised in the presence of celecoxib, suggesting the key role of arachidonic acid metabolites in EVs^Hypoxic^-mediated invasiveness. These results suggest a role for arachidonic acid accumulation in triglycerides during hypoxia that can be used later on when the cells/EVs are re-exposed to oxygen and the COX enzymes are functional. These findings offer further support to therapeutic options based upon targeting the metabolism of bioactive lipids.

Androgen and androgen receptor (AR) signaling play key roles in PCA growth and progression. In fact, androgen ablation (surgical and/or chemical castration) is a key component of PCA therapy. Importantly, androgen ablation by castration induces hypoxia caused by reduced blood flow to the prostate tissue [43, 44]. Similarly, anti-androgen casodex treatment increased the expression of hypoxia signature genes in LNCaP cells [45]. Mitani *et al.* reported that hypoxia enhances AR transcriptional activity under low androgen conditions, mimicking the androgen independence stage [46]. We have previously reported that transcriptional regulators of lipogenesis (SREBP1/2) play important role in achieving androgen-independent growth in LNCaP cells [34]. Results from the present study suggest that hypoxia promotes lipid accumulation in PCA cells independent of their AR status as we observed similar results in three different PCA cell lines- androgen-dependent LNCaP cells with functional androgen receptor (AR), androgen-independent C4-2B cells with functional AR and androgen-independent DU145 cells lacking functional AR. Together, these studies suggest that there is an intricate connection between hypoxia, androgen-AR signaling and lipogenesis, but more studies are needed to understand the key molecular mechanism for lipid accumulation under hypoxic conditions and whether hypoxia promotes androgen-independent PCA growth through enhancing lipogenesis and androgen biogenesis.

Altogether, our results show that hypoxia in PCA cells induces a signaling profile conducive to the generation of lipid synthesis and storage, a phenotype that is also reflected in the EVs they produce. The molecular lipid signature of PCA cells and their EVs may serve as a biomarker to assess the oxygenation status and aggressiveness of malignant tumors. Targeting the machinery that generates triglyceride-loaded EVs and/or the lipid-processing enzymes of the recipient cells offers potential therapeutic opportunities against PCA for the future.

## MATERIALS AND METHODS

### Cell lines and reagents

Human PCA LNCaP, DU145, 22Rv1, and C4-2B cells were purchased from ATCC (Manassas, VA). LNCaP-vehicle control (LNCaP-VC) and CPT-knockdown cells (LNCaP-CPT-KD) were earlier generated with Sigma TRCN0000036279 (CPT-KD) and the non-targeting control SHC002 purchased from the Functional Genomics Core (Boulder, CO) [30]. Preparation of the lentiviral particles was done as described [29]. RPMI1640 medium and other cell culture materials were from Invitrogen Corporation (Gaithersburg, MD). Delipidized fetal bovine serum was from Gemini Bio-products (West Sacramento, CA). ExoQuick^TM^ and exosome-free FBS (Exo-FBS^TM^) were from System Biosciences (Mountain View, CA). Antibodies for HIF1α, HIF1β, phosphorylated and/or total ACC, ACLY, hexokinase (I and II), PKM2, FASN, ACSL1, AceCS1, SCD1, mTOR, Akt, AMPK, and anti-rabbit peroxidase-conjugated secondary antibody were from Cell Signaling (Beverly, MA). Antibody for α-tubulin was from Lab Vision Corporation (Fremont, CA). ECL (Enhanced Chemiluminescence) detection system and anti-mouse HRP-conjugated secondary antibody were from GE Healthcare (Buckinghamshire, UK). Etomoxir, celecoxib, fatostatin and silibinin were from Sigma (St Louis, MO). All other reagents were obtained in their commercially available highest purity grade.

### Cell culture and hypoxia exposure

LNCaP, C4-2B, and 22Rv1 cells were cultured at 37°C in a 5% CO2 humidified environment as adherent monolayer in RPMI1640 medium supplemented with 10% fetal bovine serum (FBS) and 100 U/ml penicillin G and 100 μg/ml streptomycin sulfate. DU145 cells were cultured similarly, however 10% heat-inactivated FBS was used. LNCaP-VC and LNCaP-CPT-KD cells were cultured in LNCaP media with puromycin (1 μg/ml). Hypoxia experiments were performed using a hypoxia chamber from Biospherix (Lacona, NY) at 1% O_2_ at 37°C in a 5% CO_2_ humidified environment. For EVs isolation, LNCaP cells were cultured in media supplemented with exosome-depleted FBS. Exosomes were depleted from delipidized FBS by ultracentrifugation at 30,000 rpm for 2 hrs similar to as described below.

### EV isolation

EVs were isolated from the conditioned media following our earlier published method [2]. Briefly, LNCaP cells were cultured for 48 hrs; thereafter, media was replaced with RPMI1640 supplemented with 10% exosome-depleted FBS and cultured under normoxic (21% O_2_) or hypoxic (1% O_2_) conditions for 48 hrs. Subsequently, conditioned media was harvested and EVs were isolated by traditional methods using serial centrifugation at low speed, followed by ultracentrifugation (L-80 Ultracentrifuge, Beckman Coulter) at 30,000 rpm using type 70.1 Ti fixed angle rotor (Beckman Coulter). The EVs were also isolated by a precipitation method using commercially available Exoquick^TM^ reagent (System Biosciences) according to the vendor's instructions. Briefly, conditioned media was overnight incubated with Exoquick^TM^ reagent, centrifuged at 5,000 rpm for 2 hrs and the pellet was washed once with PBS, and pelleted EVs were resuspended in PBS and stored at −20°C until further use. EVs collected from normoxic and hypoxic PCA cells conditioned media were labelled as EV^Normoxic^ and EV^Hypoxic^, respectively.

### Lipid extraction, fractionation and analysis by gas chromatography-mass spectroscopy (GC-MS)

LNCaP cells were grown in 10 cm dishes and exposed to hypoxia for 48 hrs. Cells and EVs were resuspended in 1:1 volumes of PBS and methanol and kept at −80°C until ready to use. An aliquot of 100 μL was used for cell count using a hemocytometer for cell and EVs normalization. Lipids were extracted using the Bligh and Dyer method [47]. Samples were shaken on a rotational mixer for 1.5 hrs at 4°C, and then spun at 3,000 rpm for 15 min to separate phases. The organic bottom layer was dried down under N2 at 40°C, resuspended in chloroform, and added to aminopropyl solid phase extraction (SPE) columns (Supelclean LC-NH2, 3 ml, Supelco Analytical). Phospholipids, triglycerides and diacylglycerols were isolated using SPE based on the methods originally described by Kaluzny [48]. Lipid fractions were converted to fatty acid methyl esters (FAME) by transmethylation using sodium methoxide. Concentration and composition analysis were performed on an HP 6890 gas chromatography system with a 30m DB-23 capillary column, connected to a HP 5973 mass spectrometer. Peak identities were determined by retention time and mass spectra compared to standards of known composition.

### Western blotting

PCA cells grown under normoxic and hypoxic conditions were lysed with lysis buffer (10 mmol/L Tris-HCl, pH 7.4, 1% SDS and 2 mmol/L sodium orthovanadate), boiled in a water bath for 5 min and purge with a 27 ¼ gauge needle. EVs isolated by Exoquick^TM^ precipitation were lysed with ice-cold RIPA buffer (25mM Tris•HCl pH 7.6, 150mM NaCl, 1% NP-40, 1% sodium deoxycholate, 0.1% SDS, 0.3 mmol/L phenylmethylsulfonyl fluoride and 5 units/mL aprotinin). The protein concentration of lysates was estimated using Bio-Rad DC protein assay kit (Bio-Rad, Hercules, CA). Samples were subjected to SDS-PAGE on 8-16% tris-glycine gels and blotted onto nitrocellulose membranes. Membranes were probed with specific primary antibodies over-night at 4°C followed by peroxidase-conjugated appropriate secondary antibody for 1 hr at room temperature, and visualized by ECL detection system. Membranes were also stripped and re-probed again for protein of interest.

### Oil red o (ORO) staining

At the end of desired treatment, cells were fixed in 10% buffered formalin for 15 min at room temperature, washed twice with PBS and then with 60% isopropanol for 5 min. After isopropanol wash, the plate was completely dried and stained with ORO stain (0.3% ORO in 100% isopropanol, diluted with distilled water in the ratio of 3:2) for 30 min. After staining, cells were washed with distilled water to get a clear background. Pictures were captured at 200X magnification under a microscope and lipid content quantitation was carried out by dye elution using 100% isopropanol and absorbance was measured by spectrophotometer at 500 nm.

### Analysis of ROS levels

LNCaP cells were seeded at 50,000 cells/well in a 6 well plate. After 36 hrs, media was changed and cells were either transferred to hypoxia or continued under normoxia. After 48 hrs, cells under hypoxic condition were transferred to normoxia and etomoxir (150 μM) was added. After 24 hrs, cells were incubated with 10 μM DCF-DA for 1 hr at 37°C. The excess probe was washed off with PBS. The cells were trypsinized, resuspended in PBS and analyzed for DCF fluorescence by flow cytometry.

### Clonogenic assay

PCA cells were seeded in 6-well plates (500 cells/well). After 24 hr of plating, one set of plate was transferred to hypoxic condition (1% O_2_), while other set was maintained under normoxia. After 48 hrs, the plate under hypoxia was returned to normoxic conditions. Subsequently plates were treated with 0, 10, 25 and 50 μM of celecoxib and cultured for another 6 days, after which the cells were fixed with ice-cold methanol: glacial acetic acid (3:1) for 10 min; and stained with 1% crystal violet for 10 min. Number of colonies with greater than 50 cells were counted in each of the treatment group.

### Invasion assay

Invasion assay was performed using matrigel coated trans-well chambers from BD Biosciences (San Jose, CA). LNCaP cells were incubated under normoxic (21% O_2_) or hypoxic (1% O_2_) conditions for 48 hrs. Thereafter, to compare invasiveness, LNCaP cells were collected and seeded in the upper chambers (1×10^5^ cells per well) in RPMI medium (with 0.5% FBS) with or without celecoxib. In this assay, bottom chambers were filled with RPMI medium with 10% FBS. After 24 hrs of incubation under standard culture conditions, LNCaP cells on top surfaces of the membrane (non-invasive cells) were scraped with cotton swabs and cells on the bottom sides of the membrane (invasive cells) were fixed with cold methanol, stained with hematoxylin/eosin and mounted. Images were captured using Cannon Power Shot A640 camera on Zeiss inverted microscope and invasive cells were counted at 100x. In another experiment, LNCaP cells seeded in the upper chambers (1×10^5^ cells per well) in RPMI medium (with 0.5% FBS) with or without EV^Normoxic^ and EV^Hypoxic^ (25 μg each) and celecoxib (25 μM), and their invasiveness was determined as described above.

### Nanoparticle tracking analysis (NTA)

EVs concentration was measured using Nanosight LM10 system (Nanosight Ltd, Navato, CA) equipped with a blue laser (405 nm). EVs were illuminated by the laser and their movement under Brownian motion was captured for 60 seconds and the video recorded was subjected to NTA using the Nanosight particle tracking software to calculate nanoparticle concentrations and size.

### Statistical analysis

All values are presented as mean±SEM (*n* = 3), unless mentioned otherwise. Differences in parameters between treatments were assessed by ANOVA tests followed by Tukey post-hoc analysis. A *p* value of ≤ 0.05 was accepted as statistically significant.

## Abbreviations

ACC: Acetyl Co-A carboxylase
AceCS1: Acetyl-CoA synthetase 1
ACLY: ATP-citrate lyase
ACSL: Acyl CoA synthase ligase
AMPK: AMPactivated protein kinase
COX2: Cyclooxygenase 2
CPT1: Carnitine palmitoyltransferase 1
DCF-DA: 2′,7′ -dichlorofluorescein diacetate
DMSO: Dimethyl sulfoxide
ECL: Enhanced chemiluminescence
EV: Extracellular vesicle
FASN: Fatty acid synthase enzyme
FS: Fatostatin
HIF: Hypoxia inducible factor
mTOR: Mammalian target of rapamycin
NTA: Nanoparticle tracking analysis
ORO: Oil red O
PCA: Prostate cancer
PKM2: Pyruvate kinase 2
SCD1: Stearoyl-CoA desaturase 1
SB: Silibinin
SREBP: Sterol regulatory element binding protein
